# Cilastatin Preconditioning Attenuates Renal Ischemia-Reperfusion Injury via Hypoxia Inducible Factor-1α Activation

**DOI:** 10.3390/ijms21103583

**Published:** 2020-05-19

**Authors:** Yu Ah Hong, So Young Jung, Keum Jin Yang, Dai Sig Im, Kyung Hwan Jeong, Cheol Whee Park, Hyeon Seok Hwang

**Affiliations:** 1 Division of Nephrology, Department of Internal Medicine, The Catholic University of Korea, Seoul 06591, Korea; amorfati@catholic.ac.kr (Y.A.H.); cheolwhee@catholic.ac.kr (C.W.P.); 2 Clinical Research Institute, Daejeon St. Mary‘s Hospital, Daejeon 34943, Korea; syzzim84@gmail.com (S.Y.J.); nadia@cnu.ac.kr (K.J.Y.); 3 Department of Chemistry, College of Natural Sciences, Soonchunhyang University, Asan 31538, Korea; dsim@sch.ac.kr; 4 SH Company, 507, SCH BIT Business Incubator B/D, 22 Soonchunhyang-ro, Shinchang, Asan, Chungnam 31538, Korea; 5 Division of Nephrology, Department of Internal Medicine, College of Medicine, Kyung Hee University, Seoul 02447, Korea; khjeong@khu.ac.kr

**Keywords:** cilastatin, hypoxia inducible factor-1-α, ischemia-reperfusion injury

## Abstract

Cilastatin is a specific inhibitor of renal dehydrodipeptidase-1. We investigated whether cilastatin preconditioning attenuates renal ischemia-reperfusion (IR) injury via hypoxia inducible factor-1α (HIF-1α) activation. Human proximal tubular cell line (HK-2) was exposed to ischemia, and male C57BL/6 mice were subjected to bilateral kidney ischemia and reperfusion. The effects of cilastatin preconditioning were investigated both in vitro and in vivo. In HK-2 cells, cilastatin upregulated HIF-1α expression in a time- and dose-dependent manner. Cilastatin enhanced HIF-1α translation via the phosphorylation of Akt and mTOR was followed by the upregulation of erythropoietin (EPO) and vascular endothelial growth factor (VEGF). Cilastatin did not affect the expressions of PHD and VHL. However, HIF-1α ubiquitination was significantly decreased after cilastatin treatment. Cilastatin prevented the IR-induced cell death. These cilastatin effects were reversed by co-treatment of HIF-1α inhibitor or HIF-1α small interfering RNA. Similarly, HIF-1α expression and its upstream and downstream signaling were significantly enhanced in cilastatin-treated kidney. In mouse kidney with IR injury, cilastatin treatment decreased HIF-1α ubiquitination independent of PHD and VHL expression. Serum creatinine level and tubular necrosis, and apoptosis were reduced in cilastatin-treated kidney with IR injury, and co-treatment of cilastatin with an HIF-1α inhibitor reversed these effects. Thus, cilastatin preconditioning attenuated renal IR injury via HIF-1α activation.

## 1. Introduction

Renal ischemia-reperfusion (IR) injury is a major cause of acute kidney injury [1]. Acute ischemic injury produces excessive apoptotic cell death, and several studies have explored various stimuli to reduce injury processes [2,3]. Hypoxia-inducible factor-1α (HIF-1α) is the master regulator of cell response to hypoxia [4]. It increases the expression of several genes, including angiogenic growth factors, erythropoietin, and nitric oxide synthases [5,6,7]. Activation of these genes enhances adaptation to hypoxia and improves cell survival. Therefore, it is reasonable that HIF-1α activation before IR injury might exhibit protective effects.

Cilastatin is a molecule designed to inhibit brush border–sorted dehydrogenase peptide-1 (DHP-1). In current clinical settings, cilastatin is used to prevent the hydrolysis of antibiotics and decrease antibiotic-induced nephrotoxicity [8]. Previous in vitro and in vivo experimental studies have demonstrated that cilastatin has antioxidant and anti-apoptotic effects in drug-induced nephropathy, such as cisplatin, calcineurin inhibitor, and vancomycin [9,10,11,12]. These effects of cilastatin were associated with reduced accumulation of drug within the kidney and renal proximal tubular epithelial cells. However, the cilastatin effect is rarely investigated in the non-pharmacological renal injury. Moreover, it is unclear that cilastatin exhibits protective effects on kidney injuries.

Cilastatin binds to lipid raft in which DHP-1 is embedded. The lipid raft acts as a major platform for signaling regulation and controls Akt signaling pathways [13,14]. The Akt/mammalian target of rapamycin (mTOR) pathway is potent regulator of HIF-1α expression at translational or transcriptional level [15,16]. Therefore, cilastatin treatment can modulate the HIF-1α activity via lipid raft-related signaling pathway. However, it is less known that the cilastatin as a preconditioning stimulus activates HIF-1α pathway and the underlying mechanism of cilastatin treatment is not evaluated in the renal IR injury.

Therefore, we investigated whether preconditioning with cilastatin exhibits renoprotective effects in a mouse model of IR injury, and whether cilastatin is effective in preventing proximal tubular cell death after IR injury. We hypothesized that cilastatin treatment activates HIF-1α signaling pathway, which leads to protective effects against renal IR injury.

## 2. Results

### 2.1. Cilastatin Upregulates HIF-1α and Its Downstream Effector in HK-2 Cells

**Figure 1:**
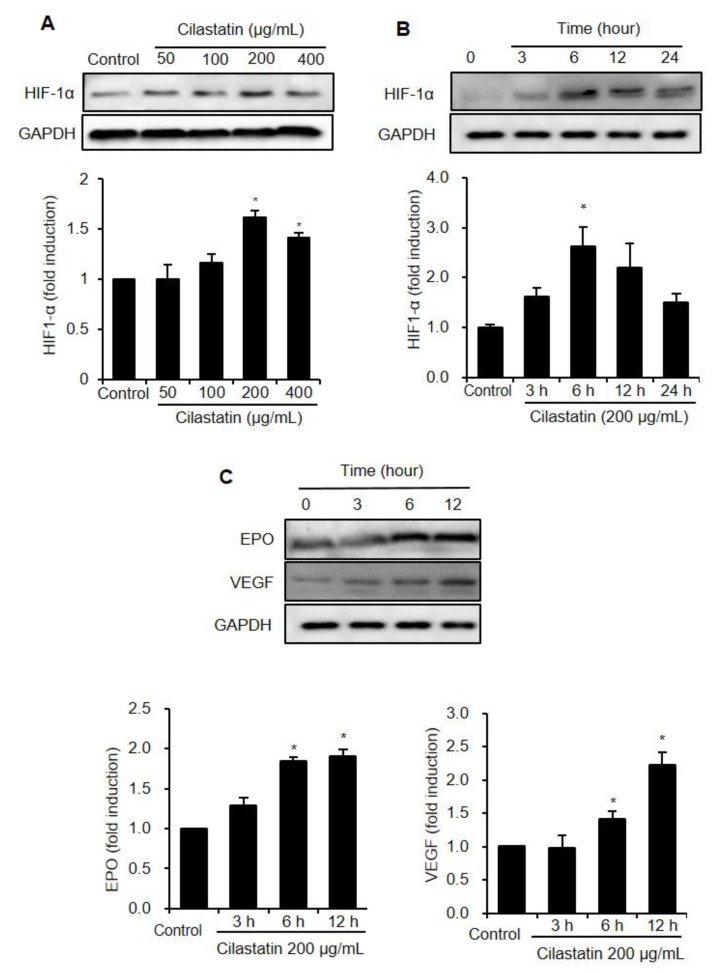
Cilastatin treatment upregulated the expressions of HIF-1α and its downstream pathway in HK-2 cells. (**A**) Semiquantitative immunoblotting revealed upregulation of HIF-1α expression by cilastatin treatment in a dose-dependent manner. (**B**) Semiquantitative immunoblotting revealed upregulation of HIF-1α expression by treatment with 200 µg/mL cilastatin in a time-dependent manner. (**C**) The expressions of VEGF and EPO proteins, determined by semiquantitative immunoblotting, were significantly elevated by treatment with 200 µg/mL cilastatin in a time-dependent manner as compared to untreated control. The data are presented as means ± SEM. * *p* < 0.05 vs. control.

Figure 1 shows the effects of cilastatin on HIF-1α expression in HK-2 cells. The expression of HIF-1α was significantly increased after cilastatin treatment in a dose- and time-dependent manner (Figure 1A,B). However, the HIF-1α mRNA level was not affected in cilastatin-treated HK-2 cells (Figure S1). Downstream effectors of HIF-1α, such as erythropoietin (EPO) and vascular endothelial growth factor (VEGF), were significantly upregulated after cilastatin treatment, respectively (Figure 1C).

### 2.2. Cilastatin Upregulates HIF-1α and Its Downstream Effector in HK-2 Cells

**Figure 2:**
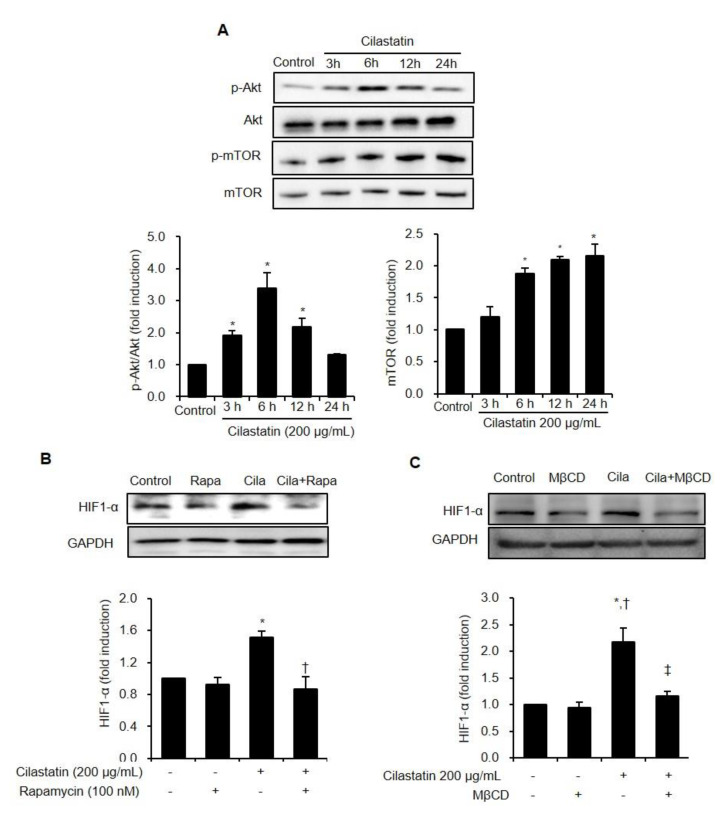
Cilastatin treatment induced HIF-1α expression via Akt/mTOR dependent pathway in HK-2 cells. (**A**) Semiquantitative immunoblotting revealed increase in Akt and mTOR expression by treatment with 200 µg/mL cilastatin in a time-dependent manner. The data are presented as means ± SEM. ^*^p < 0.05 vs. control. (**B**) Cilastatin pretreatment increased HIF-1α expression and the treatment of rapamycin, an mTOR inhibitor, significantly decreased HIF-1α expression despite cilastatin pretreatment. The data are presented as means ± SEM. * *p* < 0.05 vs. control, ^†^ *p* < 0.05 vs. rapamycin, and ^‡^*p* < 0.05 vs. cilastatin. (**C**) Cilastatin treatment increased HIF-1α expression and the destruction of lipid raft by MβCD significantly decreased HIF-1α expression despite cilastatin treatment. The data are presented as means ± SEM. * *p* < 0.05 vs. control, ^†^ *p* < 0.05 vs. MβCD, and ^‡^ *p* < 0.05 vs. cilastatin.

We studied the involved phase of protein synthesis for HIF-1α in HK-2 cells. The Akt/mTOR pathway was evaluated to assess the upstream signaling of HIF-1α, which translated the HIF-1α protein. The phosphorylation of Akt was significantly increased with maximal expression occurring at 6 h after cilastatin treatment. The phosphorylation of mTOR was also enhanced in a time-dependent manner (Figure 2A). To confirm whether HIF-1α upregulation was dependent on Akt/mTOR pathway, we exposed the cells to cilastatin in the presence of rapamycin, an mTOR inhibitor. Cilastatin increased HIF-1α expression, and co-treatment of rapamycin with cilastatin significantly reversed the HIF-1α upregulation (Figure 2B).

We conducted further experiments to investigate whether HIF-1α upregulation is dependent on lipid raft, because lipid raft modulates p-Akt/Akt signaling pathways [13,14]. The disruption of lipid raft with methyl-β-cyclodextrin (MβCD) significantly suppressed the HIF-1α expression when cilastatin increased HIF-1α expression (Figure 2C).

### 2.3. PHD/VHL-Independent Ubiquitination Pathway is Involved in Cilastatin-Mediated HIF-1α Upregulation in HK-2 cells

**Figure 3:**
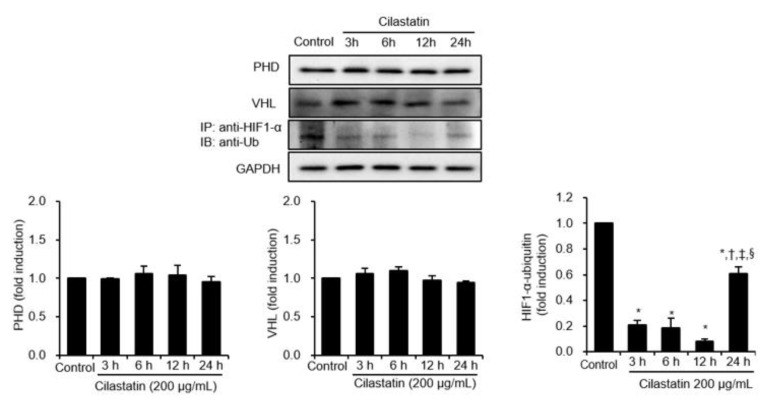
Cilastatin preconditioning upregulated HIF-1α expression via the inhibition of ubiquitination in HK-2 cells. Semiquantitative immunoblotting showed that cilastatin pretreatment did not affect the levels of VHL and PHD expressions. Immunoprecipitation showed that HIF-1α/ubiquitin complex formation was significantly suppressed in cilastatin-treated HK-2 cells compared to control in a time-dependent manner, but it was significantly increased at 24 h after cilastatin pretreatment. The data are presented as means ± SEM. *p < 0.05 vs. control, ^†,‡,§^ *p* < 0.05 vs. 3 h, 6 h, and 12 h after cilastatin treatment.

To identify whether cilastatin preconditioning activates HIF-1α by impairing its degradation pathway, the expression levels of prolyl hydroxylase domain (PHD) and von Hippel-Lindau (VHL) protein were evaluated. Cilastatin did not significantly alter the expression of PHD and VHL (Figure 3) compared to control. Therefore, cilastatin did not activate HIF-1α through the canonical HIF-1α degradation pathway. We further evaluated the interaction between HIF-1α and ubiquitin. In contrast to previous results, HIF-1α/ubiquitin complex formation was decreased after cilastatin preconditioning compared to control cells. These results suggested that cilastatin-induced HIF-1α activation was closely associated with decreased ubiquitination independent of PHD and VHL expression.

### 2.4. Cilastatin Preconditioning Enhances HIF-1α-Mediated Cell Survival in IR-Exposed HK-2 Cells

**Figure 4:**
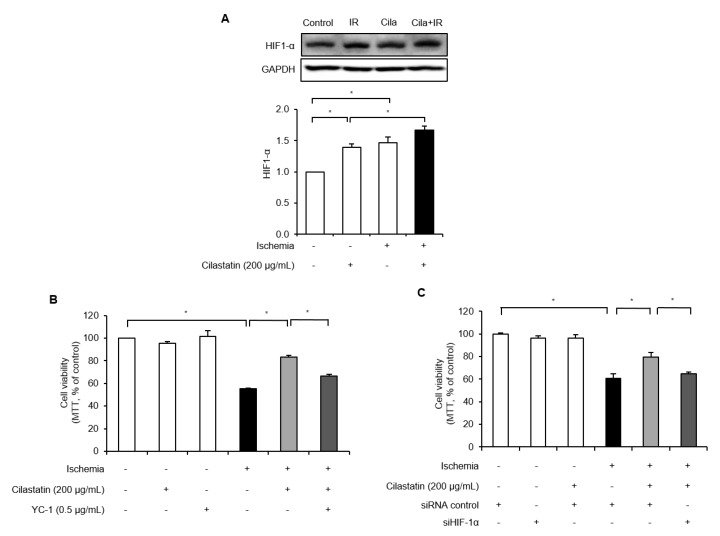
Cilastatin preconditioning protected IR-induced cell death via the activation of HIF-1α in HK-2 cells. (**A**) Semiquantitative immunoblotting of HIF-1α expression revealed that IR injury upregulated HIF-1α expression and cilastatin pretreatment further enhanced HIF-1α expression in IR-exposed cells. Cilastatin treatment also prevented IR-induced cell death. Co-treatment with cilastatin and (**B**) HIF-1α inhibitor, YC-1, or (**C**) HIF-1α siRNA restored cell death similar to those of IR-exposed cells without cilastatin treatment. The data are presented as means ± SEM. * *p* < 0.05.

We evaluated whether cilastatin preconditioning provided protection against IR injury in HK-2 cells and whether HIF-1α mediated its protective effects. Cilastatin preconditioning and IR exposure increased HIF-1α level compared with control group (Figure 4A). Cilastatin preconditioning further enhanced the HIF-1α expression in IR-exposed cells than in non-exposed cells.

IR exposure significantly reduced cell viability compared to the control group and cilastatin prevented this IR-induced cell death. The protective effect of cilastatin in IR-exposed cells was reversed by the co-treatment of YC-1, which downregulated HIF-1α at the post-translational level (Figure 4B). To evaluate whether the enhanced cell survival was associated with the specific activation of HIF-1α, we performed further experiments using HIF-1α small interfering (si) RNA. The viability of cells treated with HIF-1α siRNA was similar to that of control cells and HIF-1α siRNA treatment blocked the protective effects of cilastatin (Figure 4C).

### 2.5. Cilastatin Upregulates HIF-1α Expression Via Akt/mTOR Pathway in Mouse Kidney

**Figure 5:**
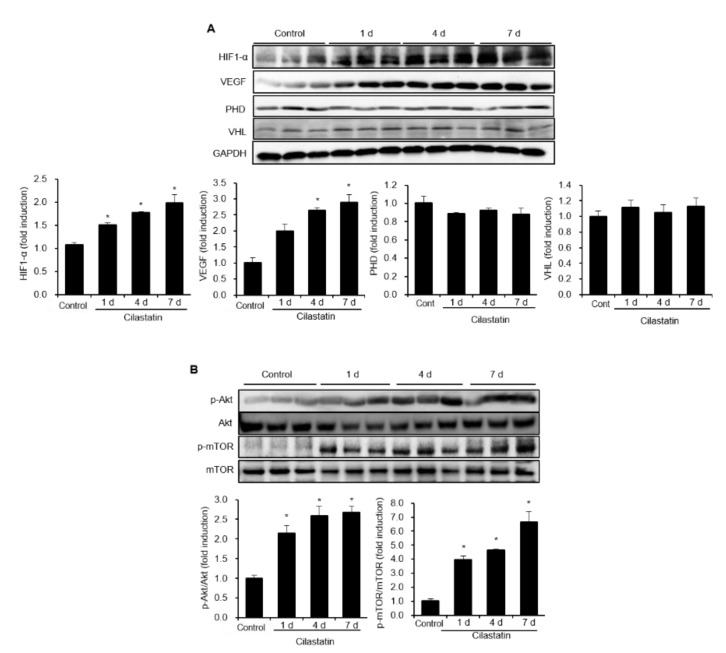
Cilastatin treatment upregulated HIF-1α expression and its upstream and downstream signaling pathways in mouse kidney. (**A**) Cilastatin treatment enhanced HIF-1α and VEGF expression in a time-dependent manner in mouse kidney, but it did not affect VHL and PHD expression. (**B**) Cilastatin treatment enhanced Akt and mTOR phosphorylation in a time-dependent manner in mouse kidney. The data are presented as means ± SEM. * *p* < 0.05.

Next, we investigated the effect of cilastatin treatment in mouse kidney. The expression of HIF-1α was significantly increased after cilastatin treatment in a time-dependent pattern (Figure 5A). The effect of cilastatin treatment on the ubiquitination pathway was evaluated in mouse kidney, and it was found that cilastatin did not affect the expressions of PHD and VHL. The expression of VEGF, which is downstream of HIF-1α, was also increased in mouse kidney. Similar to in vitro study, cilastatin treatment significantly increased the phosphorylation of both Akt and mTOR (Figure 5B).

### 2.6. Cilastatin Preconditioning Activates HIF-1α Signaling Pathway in Renal IR Injury

**Figure 6:**
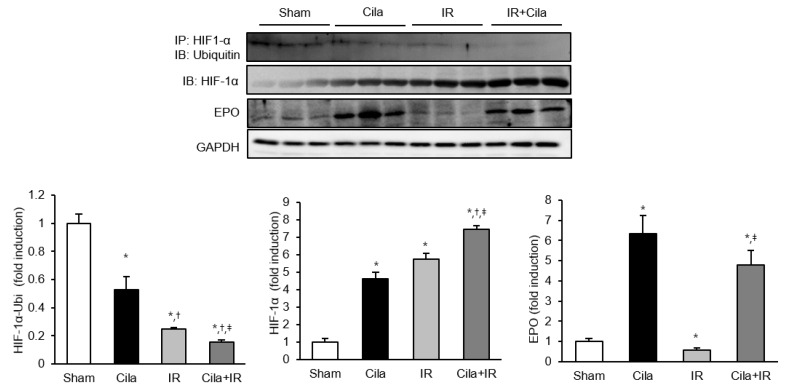
Cilastatin preconditioning upregulated HIF-1α expression and its downstream signaling pathway and decreased the ubiquitination of HIF-1α in mouse kidney with IR injury. Immunoprecipitation showed that HIF-1α/ubiquitin complex formation was significantly decreased in mouse kidney with IR injury compared to sham-operated mice; however, it more significantly decreased in cilastatin-treated mouse kidney with IR injury. Therefore, semiquantitative immunoblotting revealed that HIF-1α expression markedly increased in cilastatin-treated mouse kidney with IR injury compared to other groups. EPO expression was significantly increased in cilastatin-treated mouse kidney and was decreased in mouse kidney with IR injury; however, it increased in cilastatin-treated mouse kidney with IR injury. The data are presented as means ± SEM.* *p* < 0.05 vs. sham group; ^†^ *p* < 0.05 vs. Cila group; ^‡^ *p* < 0.05 vs. IR group.

As shown in Figure 6, HIF-1α/ubiquitin complex formation was significantly suppressed in mice with IR injury, and cilastatin preconditioning further inhibited this complex formation. HIF-1α expression in immunoblot was significantly increased in mice with IR injury, and it was further increased after cilastatin preconditioning. The expression of EPO, a downstream effector of HIF-1α, was significantly increased in sham-operated mice with cilastatin preconditioning. IR injury reduced the EPO expression in mouse kidney, which recovered in cilastatin-treated mice with IR injury.

### 2.7. Cilastatin Preconditioning Protects Against Renal IR Injury

**Figure 7:**
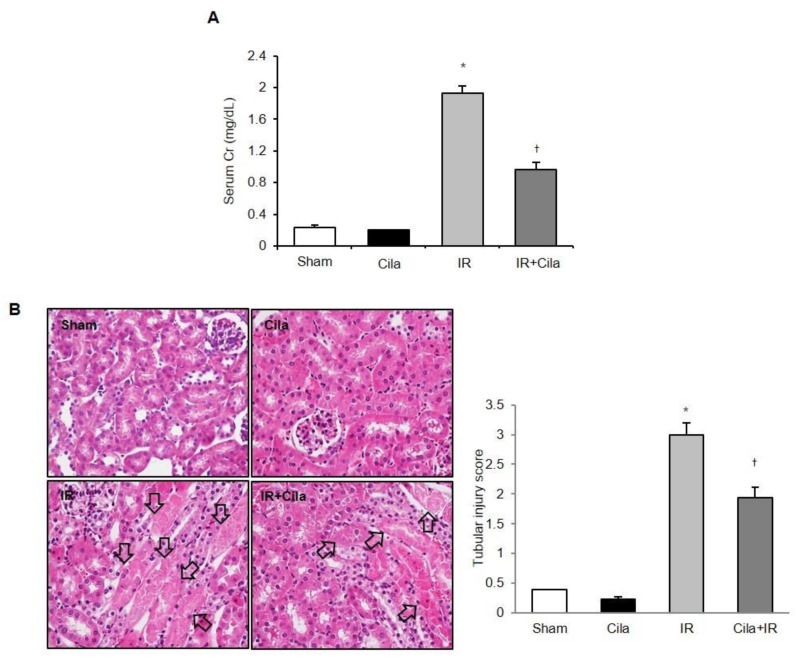
Cilastatin preconditioning improved renal function and tubular necrosis in mouse kidney with IR injury. (**A**) Serum creatinine levels were significantly increased at 24 h after IR injury compared with serum creatinine levels in sham-operated mice. Cilastatin pretreatment improved serum creatinine level in mouse kidney with IR injury. (**B**) The representative staining with hematoxylin and eosin showed a decreased tubular necrosis (arrows) in the cilastatin-treated mouse kidney with IR injury compared with mouse kidney with IR injury not treated with cilastatin (original magnification, × 200). The data are presented as means ± SEM. * *p* < 0.05 vs. sham and Cila group; ^†^ *p* < 0.05 vs. IR group.

Serum creatinine levels were significantly increased at 24 h after IR injury compared with those in sham-operated mice (Figure 7A). Cilastatin preconditioning improved serum creatinine levels compared with the mice without cilastatin preconditioning. Histologic examination of tissue sections indicated extensive tubular necrosis in the kidneys of ischemic mice compared with those of sham-operated mice (Figure 7B). Tubular necrosis was improved in the cilastatin-treated mice with IR injury compared with those not treated with cilastatin.

### 2.8. Cilastatin Preconditioning Attenuates Apoptosis in Renal IR Injury

**Figure 8:**
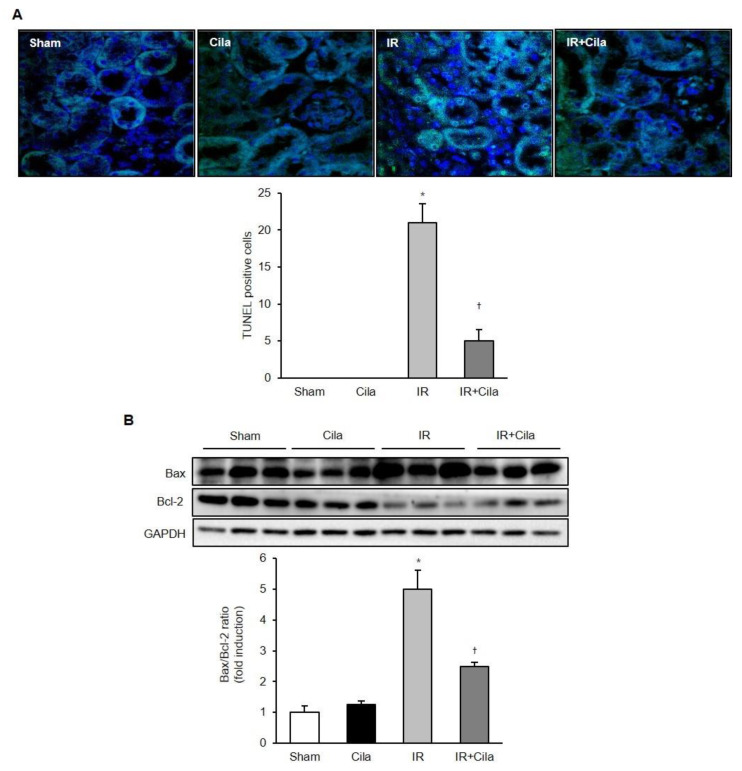
Cilastatin preconditioning attenuates apoptosis in mouse kidney with IR injury. (**A**) TUNEL assay revealed that cilastatin pretreatment significantly attenuated number of TUNEL-positive cells in mouse kidney with IR injury (original magnification, ×400). (**B**) Semiquantitative immunoblotting indicated that the level of pro-apoptotic marker, Bax, decreased in cilastatin-treated mouse kidney with IR injury, compared with mouse kidney with IR injury alone. Significant increase in levels of anti-apoptotic marker protein, Bcl-2, was noted after cilastatin pretreatment in mouse kidney with IR injury. The data are presented as means ± SEM. * *p* < 0.05 vs. sham and cila group; ^†^ *p* < 0.05 vs. IR group.

The number of terminal deoxynucleotidyl transferase-mediated dUTP nick end-labeling (TUNEL)-positive cells was increased in mice with IR injury compared to those of sham-operated mice, and it was decreased in cilastatin-treated mice with IR injury (Figure 8A). IR injury increased the expression of the proapoptotic protein Bcl-2-associated X (Bax) and decreased the expression of the antiapoptotic protein B-cell lymphoma 2 (Bcl-2). Cilastatin preconditioning significantly attenuated Bax levels and increased Bcl-2 expression in ischemic mouse kidney (Figure 8B).

### 2.9. Cilastatin Protects Against Renal IR Injury Via HIF-1α Pathway

**Figure 9:**
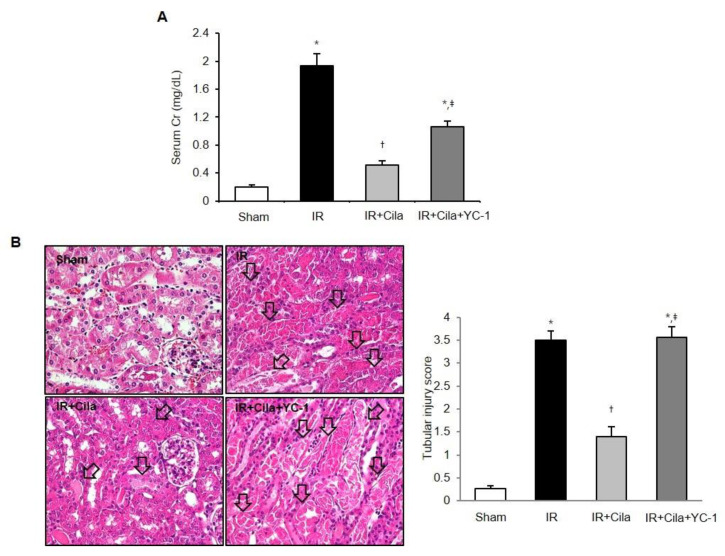
The co-treatment of YC-1, an HIF-1α inhibitor, with cilastatin worsened renal function and tubular necrosis in mouse kidney with IR injury. (**A**) Serum creatinine levels were significantly increased at 24 h after IR injury and decreased in mouse kidney with IR injury with cilastatin pretreatment. Co-treatment with cilastatin and YC-1 exacerbated renal function in mouse kidney with IR injury. (**B**) The representative staining with hematoxylin and eosin showed a decreased tubular necrosis (arrows) in the cilastatin-treated mouse kidney with IR injury, and aggravated tubular necrosis in mouse kidney with IR injury co-treated with cilastatin and YC-1 (original magnification, × 200). The data are presented as means ± SEM. * *p* < 0.05 vs. sham; ^†^ *p* < 0.05 vs. IR group; ^‡^ *p* < 0.05 vs. IR + Cila group.

Cilastatin attenuates renal dysfunction in mouse kidney with IR injury, and co-treatment with YC-1 restored IR injury to a great extent (Figure 9A). The quantitative tubular necrosis score of YC-1 co-treated ischemic mouse kidney was significantly higher than that of the mouse kidney treated with only cilastatin (Figure 9B).

## 3. Discussion

Our study demonstrated that cilastatin preconditioning induced the upregulation of HIF-1α via activation of Akt/mTOR pathway and inhibition of PHD/VHL-independent ubiquitination pathway. The cilastatin-induced HIF-1α upregulation prevented proximal tubular cell death during IR injury. In mouse kidney, cilastatin preconditioning again upregulated HIF-1α expression in the same fashion and the activated HIF-1α pathway suppressed renal dysfunction, tubular damage, and apoptotic cell death after IR injury. These findings suggested that cilastatin preconditioning exhibits protective effects against renal IR injury via HIF-1α activation. 

Preconditioning refers to exposure to a stimulus to protect organs or tissues before subjection to ischemic injury, and HIF-1α has been implicated as an attractive target pathway for ischemic preconditioning for prevention against acute kidney injury [17,18]. Our study demonstrated that cilastatin preconditioning increased the expression of HIF-1α protein and enhanced its downstream pathway. In addition, the destruction of lipid raft blocked cilastatin-induced HIF-1α expression. These findings suggested that cilastatin effectively activates HIF-1α signaling pathway and that cell membrane structure having an affinity with cilastatin is important to activate a preconditioning target.

HIF-1α is mainly located in proximal tubular cells and can be upregulated at transcriptional or translational level [17,19]. Therefore, we investigated the mRNA level of HIF-1α and Akt/mTOR pathway. We found that phosphorylated Akt/mTOR level was abundant after cilastatin preconditioning and the inhibition of mTOR pathway reduced the HIF-1α expression in cilastatin-treated HK-2 cells. However, cilastatin preconditioning did not increase the HIF-1α mRNA level in HK-2 cells. These findings demonstrated that cilastatin induces the expression of HIF-1α at the translational level, not at the transcriptional level.

HIF-1α expression can be enhanced via the enhanced Akt/mTOR pathway or by impairment of ubiquitin-proteasome degradation pathway [20,21]. Under normoxic conditions, PHD enzymes hydroxylated a subunit of HIF-1α and VHL captured them to undergo ubiquitin-protease pathway resulting in HIF-1α degradation [21,22]. However, ubiquitination of HIF-1α is also modulated via an oxygen/PHD/VHL-independent pathway involving the p53, glycogen synthase kinase 3, and the molecular chaperone 90 kDa heat-shock proteins [23,24,25]. In the present study, we demonstrated that the expressions of PHD and VHL were not altered in both HK-2 cells and mouse kidney after cilastatin preconditioning. On the other hand, immunoprecipitation analysis showed that the interactive binding between HIF-1α and ubiquitin was significantly decreased in cilastatin-treated cells under normoxic condition. Furthermore, the interaction between HIF-1α and ubiquitin was significantly decreased in mouse kidney with IR injury, and was further suppressed in cilastatin-treated mouse kidney with IR injury. These findings suggested that cilastatin may suppress HIF-1α degradation via the PHD and VHL-independent ubiquitin pathway in both normoxic and hypoxic condition.

Upregulation of renal HIF-1α plays an important role in the protection against IR injury and several studies reported HIF-1α as a potential therapeutic target [26,27]. Our study demonstrated that IR injury increased the expression of HIF-1α in proximal tubular cells and mouse kidney, and cilastatin preconditioning further increased the expression of HIF-1α. In addition, HIF-1α siRNA transfection or co-treatment with an YC-1 significantly reversed the protective effects of cilastatin in terms of proximal tubular cell death, renal dysfunction, and tubular necrosis. These data suggested that activation of HIF-1α signaling pathway plays a pivotal role in the renoprotective effect of cilastatin in IR injury. 

HIF-1α regulates the adaptive response to hypoxia and other stresses by orchestrating the transcription of protective genes [17]. EPO, a representative downstream effector of HIF-1α, prevents apoptotic cell death, and promotes tubular cell regeneration during renal IR injury [28,29]. HIF-1α activation also activates the anti-apoptotic protein, bcl-2 in renal IR injury [30,31]. Our study showed that cilastatin preconditioning upregulated the expression of EPO and decreased apoptosis in mouse kidney with IR injury. These findings suggested that cilastatin-induced HIF-1α upregulation activates downstream effectors to reduce apoptosis during renal IR injury. 

There are some interesting points and limitation in this study. Nuclear factor-erythroid-2-related factor 2 (Nrf2) is a transcription factor that regulates genes encoding antioxidant and detoxifying molecules [32,33]. It is known that Nrf2 has preventive effects against drug nephrotoxicity and ischemia reperfusion injury [34,35]. Therefore, cilastatin effects on Nrf2 is the attractive target as potential protective mechanism. Furthermore, we found Akt phosphorylation was decreased at 12 and 24 hours in cilastatin preconditioning. The reduced Akt activity was simply associated with limited working time of cilastatin. Otherwise, feedback from mTOR activation might negatively regulate the Akt activity [36,37]. The phosphorylation of mTOR at 12 h and 24 h in this experiment also supports this hypothesis. Finally, preconditioning effect of cilastatin has limitation in the clinical setting, because renal damage is already underway without preconditioning patients. Therefore, further experiments on the rescue effect of cilastatin after IR injury are required to increase the clinical usefulness.

In conclusion, cilastatin preconditioning protects against renal IR injury via the HIF-1α dependent pathway. Cilastatin preconditioning upregulated the HIF-1α expression by enhancing translational efficiency involving the Akt/mTOR pathway and by suppressing PHD/VHL-independent ubiquitination pathway. Our study provided evidence of the protective effects of cilastatin in non-pharmacological renal injury and demonstrated that the wide clinical application of cilastatin could be expected to prevent acute kidney injury.

## 4. Materials and Methods 

### 4.1. Human Proximal Tubular Cell Culturing

HK-2 cells were purchased from American Type Culture Collection (Manassas, VA, USA). The cells were grown and passaged in Dulbecco’s modified Eagle’s medium (DMEM) supplemented with 10% fetal bovine serum, 50 U/mL penicillin, and 50 µg/mL streptomycin. The cells were cultivated in a humidified 5% CO2 environment at 37 °C. HK-2 cells were plated and cultured to 80% confluence.

HK-2 cells were treated with different doses of cilastatin for different times. The control cells were treated with distilled water. We also harvested the cells 24 h after co-treatment of cilastatin and MβCD (Sigma-Aldrich, St Louis, MO, USA), or mTOR inhibitor (rapamycin, Sigma-Aldrich, St Louis, MO, USA). 

HK-2 cells were placed in serum-free media for 16 h at 80% confluence and were pretreated with cilastatin, HIF-1α inhibitor, YC-1 [38], or HIF-1α siRNA (Bioneer, Daejeon, Korea) for 1 h. Scrambled siRNA were complexed with a transfection reagent (Invitrogen, Carlsbad, CA, USA). After washing with phosphate-buffered saline (PBS), they were exposed to ischemia by immersing the cellular monolayer in mineral oil (Sigma-Aldrich, St Louis, MO, USA) for 90 min [39]. Then, the cells were washed twice and then received the same treatment. 

### 4.2. Cell Viability

A commercially available MTT assay kit (EZ-Cytox; Daeil Lab Service, Seoul, Korea) determined cell viability. After exposure to ischemia and 24 h reperfusion, 10 μL of cell viability assay reagent was added. The optical densities of the samples were determined at 450 nm in a microplate reader (Bio-Rad Laboratories, Hercules, CA, USA).

### 4.3. Animal Model of Renal IR Injury

Seven- to eight-week-old male C57BL/6J mice were housed under a 12 h light–dark cycle, and food and water were freely available. Crystalline cilastatin was kindly provided by Im DS (Department of Chemistry, Soonchunhyang University, Cheonan, Korea). The experimental protocol was approved by the animal experiments’ ethics committee of Daejeon St. Mary‘s Hospital (1st February 2016, CMCDJ-AP-2016-009).

Mice were divided into five groups (sham, sham + cilastatin, IR, IR + cilastatin, and IR + cilastatin + YC-1) and each group consisted of six mice. Cilastatin was diluted in saline, and 300 mg/kg of cilastatin, with or without YC-1 (5 mg/kg/day), was intraperitoneally injected daily for seven consecutive days before ischemia induction. The sham and IR groups of mice received the same volume of saline. Renal IR injury was performed under tiletamine–zolazepam (30 mg/kg) and xylazine (10 mg/kg) anesthesia. The mice were subjected to renal IR injury using previously described methods [40,41,42]. The bilateral renal pedicles were occluded for 23 min using microvascular clamps. A homoeothermic pad maintained the core body temperature of mice. The mice were sacrificed 24 h after ischemia and tissue and blood samples were collected.

### 4.4. Functional and Morphological Changes due to Kidney Injury

Serum creatinine level was measured by an IDEXX VetTest^®^ Chemistry Analyzer (IDEXX Laboratories, Inc., Westbrook, ME, USA). The kidney tissues were fixed in 10% formalin buffer, embedded in paraffin, and then, cut into 3.5 mm-thick sections. Hematoxylin and eosin staining was performed to evaluate the degree of tubular damage. Markers of tubular damage were scored by calculating the percentage of tubules in the corticomedullary junction that displayed cell necrosis, loss of brush border, cast formation, and tubular dilation, as follows: 0, none; 1, ≤10%; 2, 11–25%; 3, 26–50%; 4, 51–75% and 5, ≥76%. The tubular necrosis score was quantified per high power field of each kidney and at least 20 fields were reviewed from each slide.

### 4.5. Immunofluorescence Staining

The number of apoptotic HK-2 cells was counted by TUNEL using a TUNEL Apoptosis Detection kit (Intergen, Purchase, NY, USA). The immunofluorescence images for TUNEL assay were captured by confocal microscopy (LSM5 Live Configuration Variotwo VRGB; Zeiss, Oberkochen, Germany). The number of positive cells was quantified per high-power field (HPF) of each kidney, and at least 20 fields were reviewed for each slide.

### 4.6. Immunoblotting Analyses of HK-2 Cells And Kidney Tissue

We performed the immunoblotting analyses for mouse kidneys and HK-2 cell lysates as described previously [42]. Kidney tissues were homogenized and resolved by SDS-polyacrylamide gel electrophoresis (SDS-PAGE) after centrifugation. HK-2 cells were harvested, washed with cold PBS, and resuspended in lysis buffer. Equal amounts of protein were electroblotted onto a nitrocellulose membrane. The membrane was blocked and incubated with primary antibodies directed against HIF-1α (Abcam, Cambridge, UK), Akt (Cell Signaling Technology, Beverly, MA, USA), pS473 Akt (Cell Signaling Technology), mTOR (Cell Signaling Technology), pSer2448 mTOR (Cell Signaling Technology), PHD (Cell Signaling Technology), VHL (von Hippel-Lindau, Santa Cruz Biotechnology), EPO (Abcam), VEGF (Abcam, Cambridge, UK), Bax (Cell Signaling Technology), Bcl-2 (Cell Signaling Technology), and glyceraldehyde-3-phosphate dehydrogenase (GAPDH, Cell Signaling Technology). They were then incubated with horseradish peroxidase-conjugated anti-rabbit IgG or anti-mouse IgG antibody (Invitrogen, Carlsbad, CA, USA). Positive bands were detected and analyzed using the ChemiDoc XRS Image system (Bio-Rad Laboratories, Hercules, CA, USA).

### 4.7. Real-Time Reverse Transcription PCR

Total RNA was isolated from kidney tissues and HK-2 cells using a NucleoSpin^®^ RNA II kit (Macherey-Nagel, Düren, Germany). cDNA was synthesized using Reverse Transcriptase Premix (Elpis Biotech, Daejeon, Korea) and amplified in a Power SYBR® Green polymerase chain reaction (PCR) Master Mix (Applied Biosystems, Warrington, UK) with gene-specific primer pairs (HIF-1α: F; 5’-TGCCCCAGATTCAAGATCAGC-3’, R; 5’-GGCTGGGAAAAGT TAGGAGTGT-3’) Quantitative real-time PCR was performed on an ABI 7500 FAST instrument (Applied Biosystems, Warrington, UK). The expression levels of mRNAs were normalized to the expression of GAPDH.

### 4.8. Immunoprecipitation

Cultured HK-2 cells and kidney tissues were lysed with kinase buffer and then 1 mg of lysate was immunoprecipitated using 1 µg of anti-ubiquitin antibody (Santa Cruz Biotechnology) and protein G Sepharose 4 Fast Flow (GE Healthcare, Danderyd, Sweden). After washing with KB without 1% NO40, immunoblotting was performed using HIF-1α antibody (Biorbyt Ltd., Cambridge, UK).

### 4.9. Statistical Analysis

Data are expressed as the mean ± standard error of the mean (SEM) of ≥3 independent experiments. Differences between the two groups were determined using Student’s t test or the Mann–Whitney U test. Multiple comparisons were performed using one-way analysis of variance and Tukey’s post hoc test. Statistical analysis was performed using SPSS software (version 22.0; IBM, Armonk, NY). Results were considered significant when *p* < 0.05.

## Supplementary Materials

Supplementary materials can be found at https://www.mdpi.com/1422-0067/21/10/3583/s1.

## Abbreviations

IR	Ischemia–reperfusion
HIF-1α	Hypoxia-inducible factor-1α
DHP-1	Dehydrogenase peptide-1
mTOR	Mammalian target of rapamycin
EPO	Erythropoietin
VEGF	Vascular endothelial growth factor
MβCD	Methyl-β-cyclodextrin
PHD	Prolyl hydroxylase
VHL	Von Hippel-Lindau
si	Small interfering
TUNEL	terminal deoxynucleotidyl transferase-mediated dUTP nick end-labeling
Nrf2	nuclear factor-erythroid-2-related factor 2
Bax	Bcl-2-associated X protein
Bcl-2	B-cell lymphoma 2
GAPDH	glyceraldehyde-3-phosphate dehydrogenase
PCR	polymerase chain reaction
