# Supplementary material for: Cilastatin Preconditioning Attenuates Renal Ischemia-Reperfusion Injury via Hypoxia Inducible Factor-1α Activation
Source: Int J Mol Sci. 2020 May 19;21(10):3583. doi: 10.3390/ijms21103583 (PMC7279043; doi:10.3390/ijms21103583)
Supplement: Supplementary file 1 [file ijms-21-03583-s001.pptx]

## Slide 1
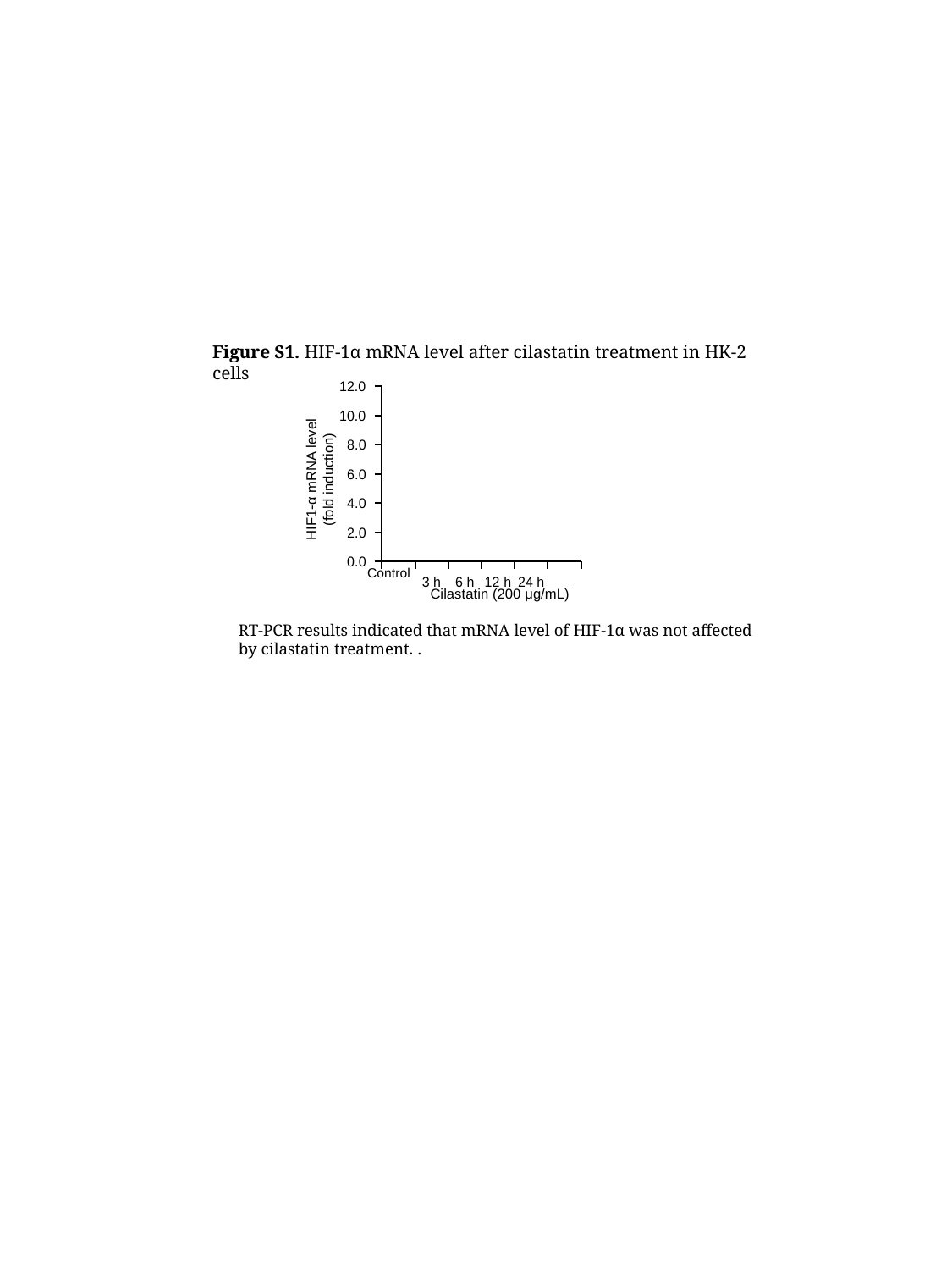

Figure S1. HIF-1α mRNA level after cilastatin treatment in HK-2 cells
### Chart
| Category | HIF1-α |
|---|---|
| | 1.0083796797408888 |
| 3 h | 0.9001928851820203 |
| 6 h | 0.9170381386996375 |
| 12 h | 0.9801197 |
| 24 h | 0.9544512786220292 |HIF1-α mRNA level (fold induction)
Control
Cilastatin (200 μg/mL)
RT-PCR results indicated that mRNA level of HIF-1α was not affected by cilastatin treatment. .
